# Recognition-Encoded Synthetic Information Molecules

**DOI:** 10.1021/acs.accounts.3c00029

**Published:** 2023-03-09

**Authors:** Giulia Iadevaia, Christopher A. Hunter

**Affiliations:** Yusuf Hamied Department of Chemistry, University of Cambridge, Lensfield Road, Cambridge CB2 1EW, U.K.

## Abstract

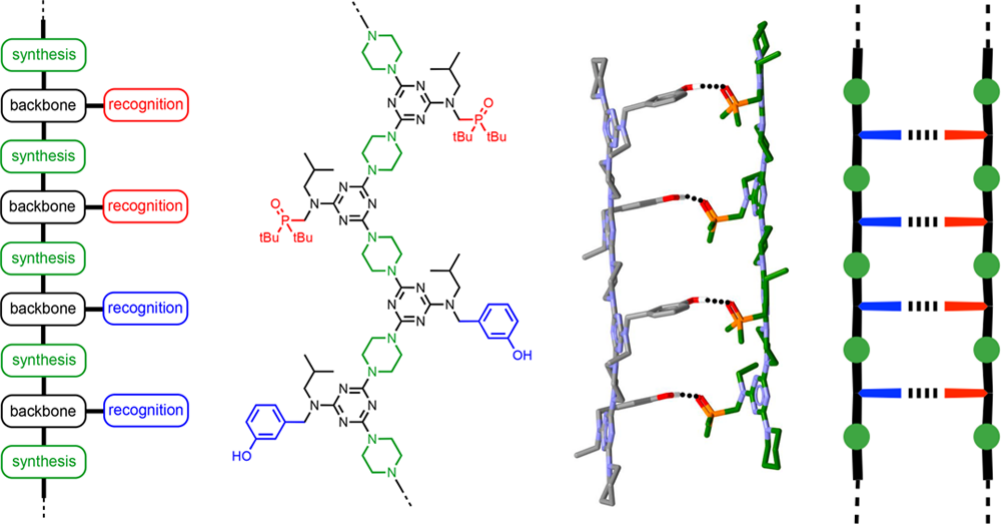

Nucleic acids represent a unique class of highly programmable molecules, where the sequence of monomer units incorporated into the polymer chain can be read through duplex formation with a complementary oligomer. It should be possible to encode information in synthetic oligomers as a sequence of different monomer units in the same way that the four different bases program information into DNA and RNA. In this Account, we describe our efforts to develop synthetic duplex-forming oligomers composed of sequences of two complementary recognition units that can base-pair in organic solvents through formation of a single H-bond, and we outline some general guidelines for the design of new sequence-selective recognition systems.

The design strategy has focused on three interchangeable modules that control recognition, synthesis, and backbone geometry. For a single H-bond to be effective as a base-pairing interaction, very polar recognition units, such as phosphine oxide and phenol, are required. Reliable base-pairing in organic solvents requires a nonpolar backbone, so that the only polar functional groups present are the donor and acceptor sites on the two recognition units. This criterion limits the range of functional groups that can be produced in the synthesis of oligomers. In addition, the chemistry used for polymerization should be orthogonal to the recognition units. Several compatible high yielding coupling chemistries that are suitable for the synthesis of recognition-encoded polymers are explored. Finally, the conformational properties of the backbone module play an important role in determining the supramolecular assembly pathways that are accessible to mixed sequence oligomers.

Almost all complementary homo-oligomers will form duplexes provided the product of the association constant for formation of a base-pair and the effective molarity for the intramolecular base-pairing interactions that zip up the duplex is significantly greater than one. For these systems, the structure of the backbone does not play a major role, and the effective molarities for duplex formation tend to fall in the range 10–100 mM for both rigid and flexible backbones. For mixed sequences, intramolecular H-bonding interactions lead to folding. The competition between folding and duplex formation depends critically on the conformational properties of the backbone, and high-fidelity sequence-selective duplex formation is only observed for backbones that are sufficiently rigid to prevent short-range folding between bases that are close in sequence. The final section of the Account highlights the prospects for functional properties, other than duplex formation, that might be encoded with sequence.

## Key References

- TroseljP.; BolgarP.; BallesterP.; HunterC. A.High-Fidelity Sequence-Selective Duplex Formation by Recognition-Encoded Melamine Oligomers. J. Am. Chem. Soc.2021, 143, 8669–86783408186410.1021/jacs.1c02275PMC8213060.^1^*This work describes the first example of high selectivity duplex formation, where information was programmed into mixed sequence oligomers using single H-bond recognition sites*.
- Núñez-VillanuevaD.; IadevaiaG.; StrossA. E.; JinksM. A.; SwainJ. A.; HunterC. A.H-Bond Self-Assembly: Folding versus Duplex Formation. J. Am. Chem. Soc.2017, 139, 6654–66622847007010.1021/jacs.7b01357PMC5469522.^2^*This work describes folding in mixed sequence oligomers, methods that can be used to quantify the competing equilibria, and the criteria for successful duplex formation*.

## Introduction

Nucleic acids are Nature’s information molecules, and the determination of the double helix structure represents a milestone in science.^3^ The information encoded in the sequence of the nucleobases can be read via sequence-selective duplex formation and copied into polymeric nucleic acids or proteins via template-directed synthesis. These unrivalled properties have been exploited to develop programmable nanostructures and to discover new functional biopolymers using directed evolution. Analogues, where the furanose sugar,^4−7^ the phosphate linker,^8−20^ or the bases^21−26^ have been replaced, also form sequence-selective duplexes, suggesting that it might be possible to develop different types of synthetic polymers that exhibit functional properties that are currently unique to nucleic acids.

**Figure 1:**
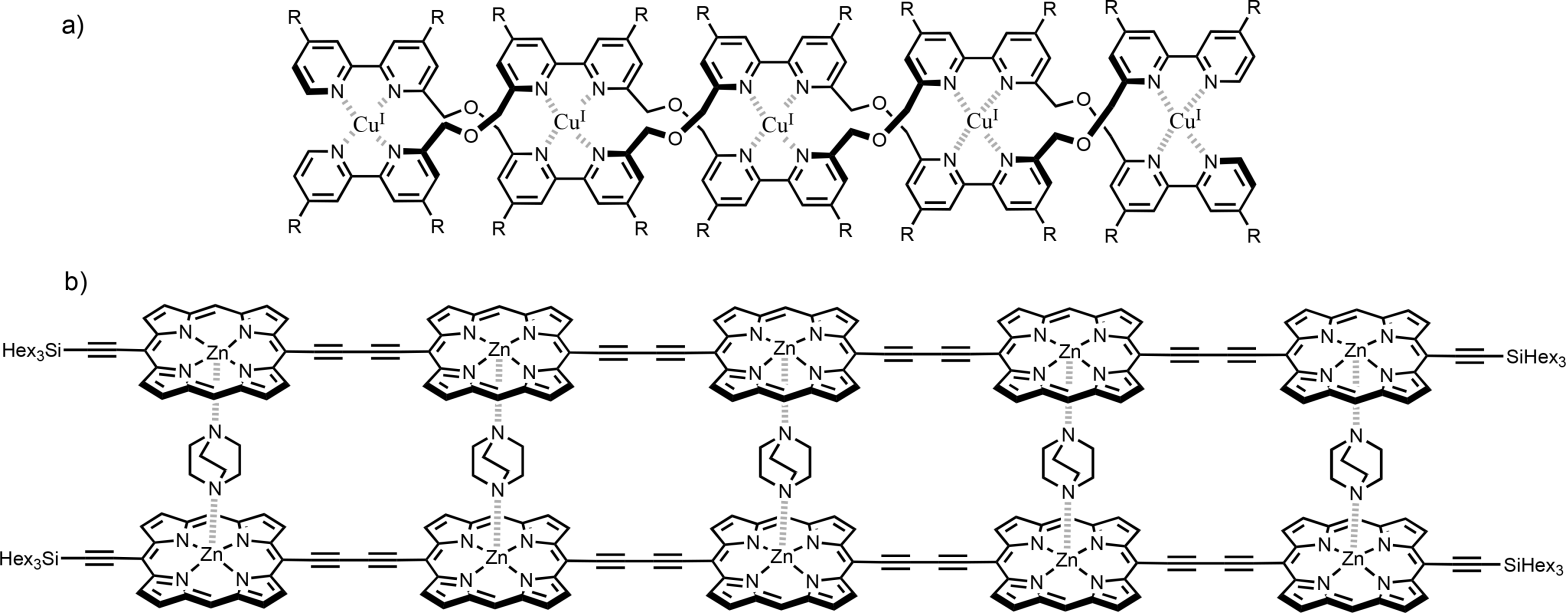
Synthetic duplexes formed by metal–ligand coordination. (a) Bipyridine oligomers form duplexes in the presence of metal ions (R = CONEt_2_). (b) Zinc porphyrin oligomers form duplexes in the presence of bidentate ligands.

**Figure 2:**
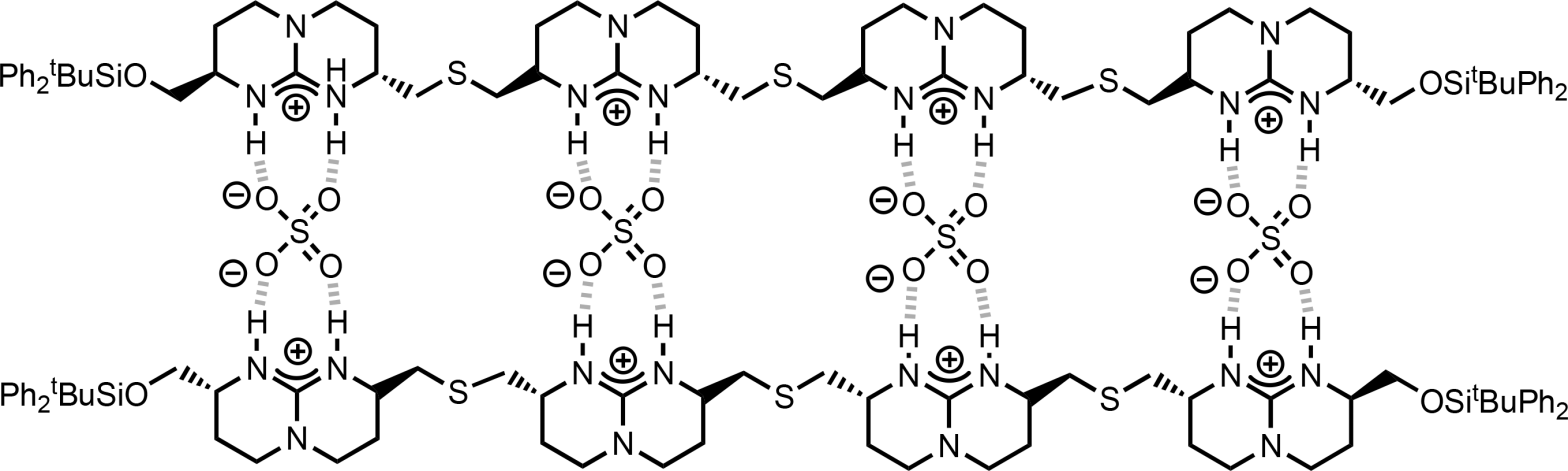
Synthetic duplexes formed by salt bridge interactions. Guanidinium oligomers form duplexes in the presence of sulfate ions.

**Figure 3:**
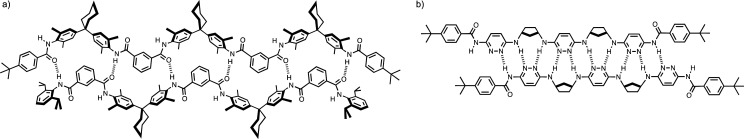
Synthetic duplexes formed by H-bonding. (a) Amide oligomers. (b) Diaminopyridazine oligomers.

Different types of noncovalent chemistry have been explored for the assembly of duplexes using synthetic oligomers: metal–ligand coordination,^27−30^ stacking,^31,32^ salt bridges,^33−37^ and H-bonding.^38−50^Figure 1a shows an oligo(2,2′-bipyridine) ligand that forms a helical duplex in the presence of metal ions.^27^ When oligomers with different numbers of bipyridine units were mixed with copper(I), selective formation of duplexes between strands of the same length was observed. Figure 1b shows a zinc porphyrin oligomer that coordinates 1,4-diazabicyclo[2.2.2]octane to form a ladder duplex.^29^ Association constants for duplexes formed by oligomers with different numbers of porphyrins were found to increase uniformly with chain length. The guanidinium oligomers in Figure 2 form duplexes in the presence of sulfate ions due to salt bridge interactions.^33^Figure 3 shows synthetic oligomers that form duplexes via H-bonding interactions. The stabilities of the duplexes formed by length-complementary oligoamides (Figure 3a)^38,39^ and diaminopyridazine oligomers (Figure 3b)^50^ both increase uniformly with the number of H-bonds.

**Figure 4:**
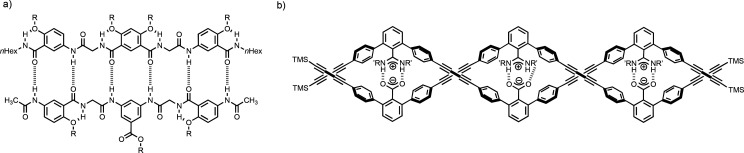
Synthetic duplexes that encode sequence information. (a) The sequence of H-bond donor and acceptor sites encodes information in oligoamides (R = *n*-octyl). (b) The sequence of charged side chains encodes information in terphenyl oligomers (R′ = 1-phenylethyl).

These early examples of synthetic duplexes were all based on homo-oligomers that did not contain any sequence information, so complementarity was based purely on length. Figure 4 illustrates two different architectures that allow the introduction of different monomer units leading to sequence-selective duplex formation. The sequence-complementary oligoamides shown in Figure 4a form a duplex that is an order of magnitude more stable than the corresponding duplexes formed by oligomers with a single mismatch.^42^Figure 4b shows oligomers that form a helical duplex via salt bridge interactions.^35^ When six different 3-mer sequences were mixed, only sequence-complementary duplexes were observed.

## Design Criteria

**Figure 5:**
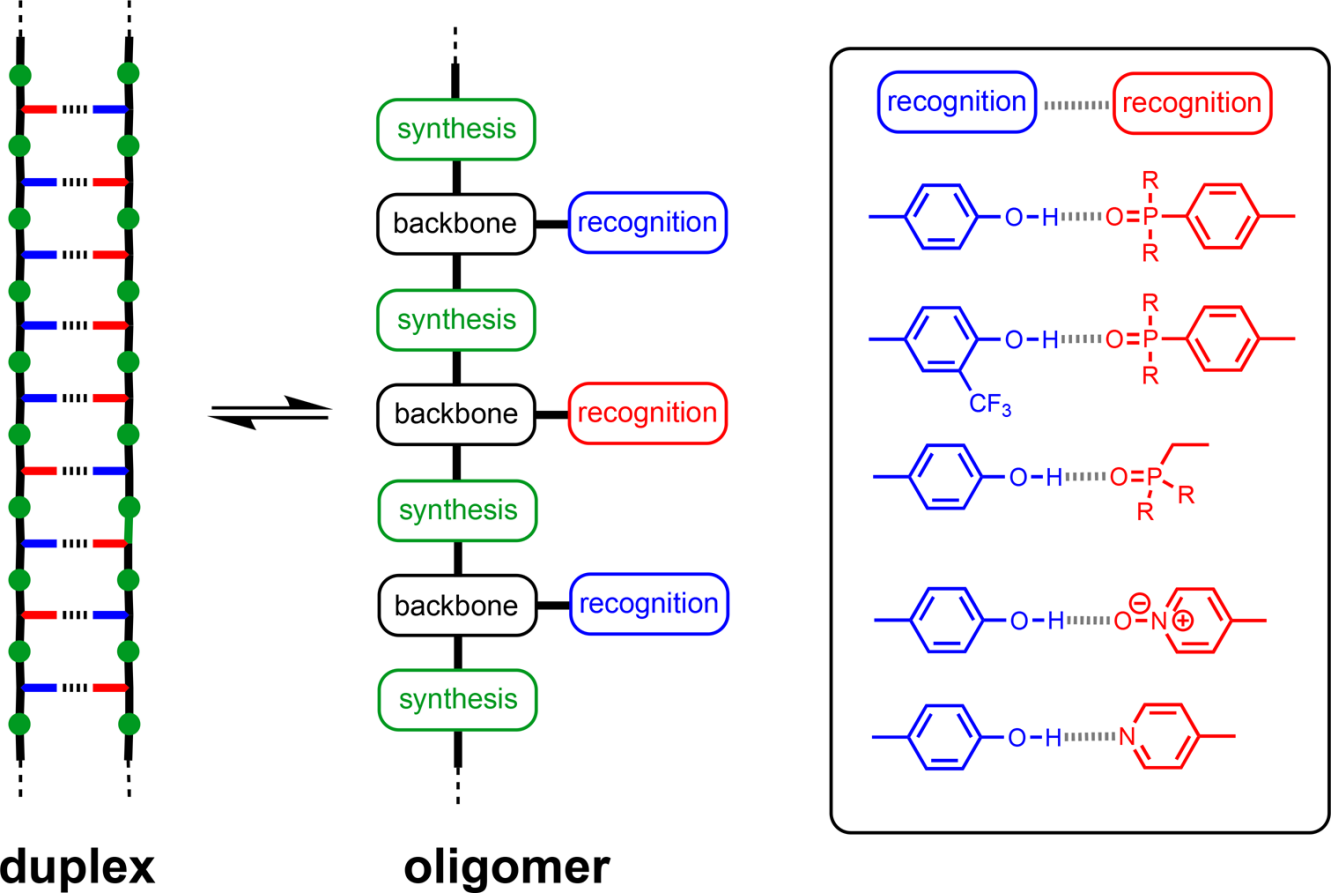
Blueprint of the structure of a synthetic information oligomer. The recognition-based pairing system (red/blue), the synthesis module (green), and the backbone (black) are the three key components of the design. Examples of single H-bond base-pairing systems that have been implemented as recognition modules are shown.

Although the oligomers described above have very different structures, most have the recognition elements embedded in the backbone, which limits the possibilities for modification without major redesign. In contrast, the modular nature of the nucleic acid architecture has allowed the development of a wide range of synthetic analogues without compromising duplex formation. We have therefore focused on the nucleic acid blueprint illustrated in Figure 5.^51^ We identify three key components: a recognition system for base-pairing; chemistry for synthesis of oligomers; a backbone module that defines the geometric complementarity. Many different chemical implementations of this basic blueprint are possible, and the approach we have taken is explained below.

### Recognition Modules

We have opted for a base-pairing system based on a single H-bond (see Figure 5), and this two-letter alphabet provides the basis for encoding sequence information. Although the information density encoded by two bases is lower than the 4-letter alphabet used in nucleic acids, the advantage is that mispairing is not an issue, because acceptors cannot H-bond to acceptors and the interaction between two phenol donors is extremely weak.

**Figure 6:**
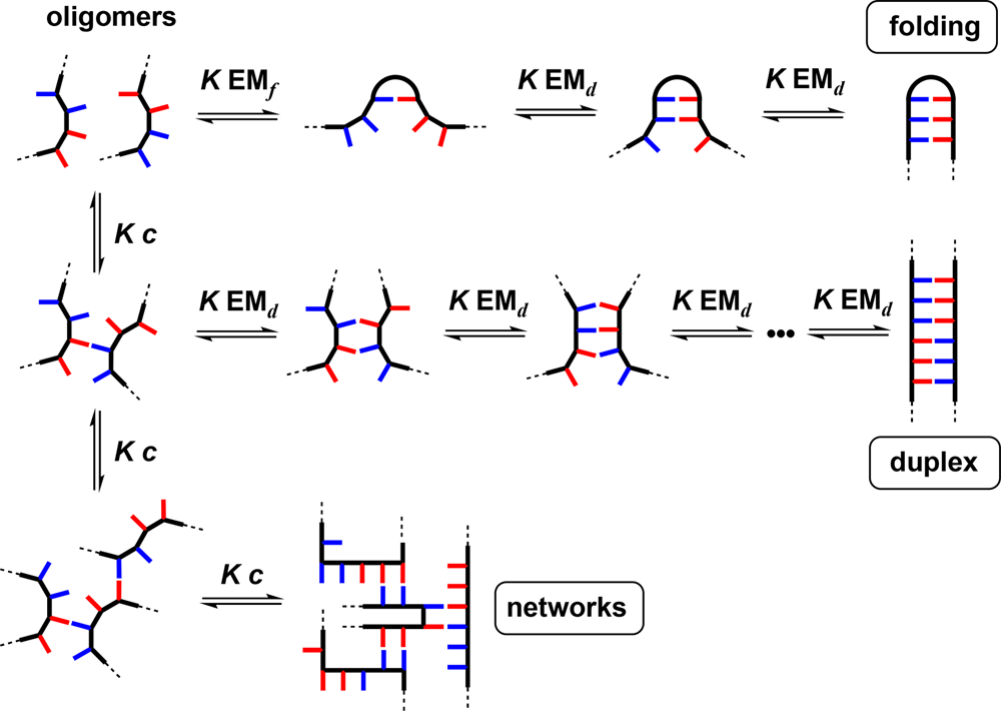
Three channels for the self-assembly of recognition-encoded oligomers and the key parameters that determine the outcome: *K* is the association constant for a single intermolecular base-pairing interaction, EM_d_ is the effective molarity for intramolecular interactions that zip up the duplex, EM_f_ is the effective molarity for intramolecular interactions that lead to loop formation and folding, and *c* is the operating concentration.

Figure 6 shows that in addition to assembly of a duplex there are other possible outcomes of the formation of base-pairs in mixed sequence oligomers: intramolecular interactions lead to folding, and intermolecular interactions lead to polymeric networks. Intermolecular polymerization ultimately leads to precipitation, and in Nature, the polyelectrolyte structure of nucleic acids helps to avoid this pathway.^24^ The key parameter that governs the duplex channel is the product *K* EM_d_, where *K* is the association constant for formation of an intermolecular base-pair and EM_d_ is the effective molarity for the intramolecular interactions that zip up the duplex. The channel leading to duplex assembly is downhill in free energy terms when *K* EM_d_ > 1. Since effective molarities for noncovalent interactions tend to fall in a narrow range (10–100 mM),^52,53^ it is possible ensure *K* EM_d_ > 1 by choosing a H-bond with *K* > 100 M^–1^ for the base-pairing interaction.

The association constant for formation of a single intermolecular H-bond can be reliably estimated using eq 1. Tabulated values of functional group H-bond parameters α and β were used in eq 1 to identify combinations of base-pairing partners and solvents that maximize the potential for duplex formation. Figure 5 shows the base-pairing systems that we have investigated, and Table 1 shows the corresponding H-bond parameters and association constants. The weakest interaction is between phenol and pyridine, and the association constants in Table 1 suggest that this base-pair is only likely to work if the value of EM_d_ is high enough to compensate. The other three base-pairs are all suitable for duplex formation in toluene (*K* > 100 M^–1^), and the 2-(trifluoromethyl)phenol·phosphine oxide base-pair should also work well in chloroform.
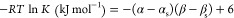
1where α and β are the H-bond parameters of the donor and acceptor functional groups and α_s_ and β_s_ are the corresponding H-bond parameters for the solvent.^54^

**Table 1:** Association Constants (*K*, M^–1^) Estimated Using Eq 1 for H-Bonding Interactions^a^

				solvent	
donor	acceptor	α	β	*K* (toluene)	*K* (chloroform)
phenol	phosphine oxide	3.8	10.7	1300	50
phenol	pyridine	3.8	7.2	25	6
phenol	pyridine *N*-oxide	3.8	9.0	190	20
2-(trifluoromethyl)phenol	phosphine oxide	4.3	10.7	7300	390

a Solvent H-bond parameters are α_s_ = 2.2, β_s_ = 0.8 for chloroform and α_s_ = 1.0, β_s_ = 2.1 for toluene.

There are some additional considerations to ensure that duplex assembly dominates over the other self-assembly channels in Figure 6. In order to minimize the polymeric network channel, EM_d_ must be greater than *c*, the operating concentration. Since EM_d_ is likely to fall in the range 10–100 mM, networks can be avoided by working at concentrations of 1 mM or lower.^53^ The folding channel can be avoided if EM_f_ ≪ EM_d_. The relative values of EM_d_ and EM_f_ depend on the conformational properties of the backbone, which makes prediction difficult, and the relationship with folding and duplex formation will be discussed below.

### Synthesis Modules

**Figure 7:**
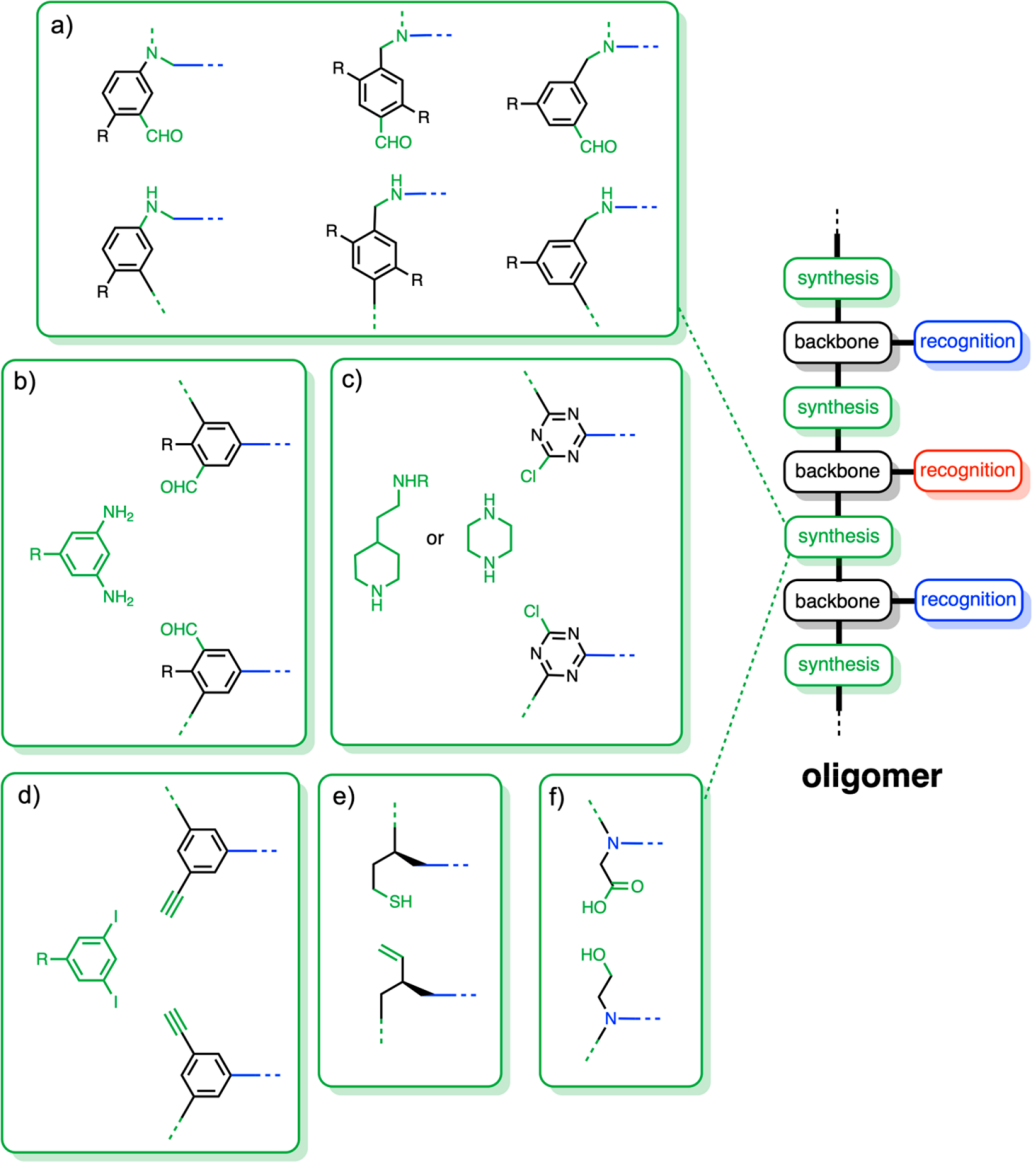
Synthesis modules used for the preparation of oligomers. (a) Reductive amination. (b) Imine condensation. (c) Nucleophilic aromatic substitution. (d) Sonogashira coupling. (e) Thiol–ene coupling. (f) Ester coupling. R represents solubilizing groups.

The coupling chemistry used for the synthesis of oligomers should be high yielding, and the reactions should not generate any polar functional groups that could compete for H-bonding interactions with the recognition modules. There are additional limitations if the coupling chemistry is to be used in template-directed synthesis: the reactions should be compatible with the functional groups used for the backbone and recognition system, and the reactions should proceed in the nonpolar solvents required for efficient base-pairing. Reductive amination, imine formation, Sonogashira coupling, the thiol–ene reaction, and Grubbs metathesis all meet these criteria. We have not yet been able to develop suitable monomers for use with Grubbs metathesis, but Figure 7 shows backbones made using the other reactions.

Reductive amination of an aromatic aldehyde with a secondary aniline was used to obtain a series of isomeric oligomers with a polyaniline backbone (Figure 7a).^2,51,55−58^ The tertiary aniline products are poor H-bond acceptors (β = 4) and do not compete with the recognition system. Imine formation from an aromatic aldehyde and a primary aniline was used to prepare oligomers, which have the potential for use in dynamic covalent chemistry or can be trapped as polyamines by reduction (Figure 7b).^59,60^ The secondary anilines in the polyamine backbone are not sufficiently strong H-bond donors (α = 2) to compete with the recognition system. Sonogashira coupling was used for the synthesis of poly(phenylacetylene) hydrocarbon backbones (Figure 7d).^61,62^ Polythioethers were obtained via thiol–ene reactions (Figure 7e), and homochiral monomer building blocks were used to obtain homochiral oligomers.^63^ The thioether linkers in the product are weak H-bond acceptors (β = 4) that do not compete with the recognition system.

We have also investigated two other coupling reactions that at first sight do not appear to meet the criteria listed above. Melamine oligomers can be obtained via nucleophilic aromatic substitution reactions of cyanuric chloride with secondary amines.^1^ This chemistry generates a backbone, which has good H-bond acceptors on the triazine rings, but these sites can be sterically blocked using alkyl substituents on the exocyclic nitrogen atoms to prevent competition with the recognition system (Figure 7c). We have also made oligomers with ester backbones.^64,65^ Ester coupling is not orthogonal to the recognition system, so protection of the phenol side chains was required for the synthesis of these oligomers (Figure 7f). Although the ester linkages in the product are slightly more polar than the functional groups present in the other backbones (β = 5), they did not compete with the recognition system.

### Solubilizing Groups

The nonpolar solvents required for efficient base-pairing mean that solubilizing groups are a necessary design element. The backbones in Figure 7a–d all incorporate sites for attachment of branched alkyl substituents, and the functional groups used to attach the solubilizing groups are relatively nonpolar (β = 3–5). The backbones in Figure 7e,f do not have solubilizing groups, which could limit the extension of these architectures to polymeric systems.

## Synthetic Strategies

**Figure 8:**
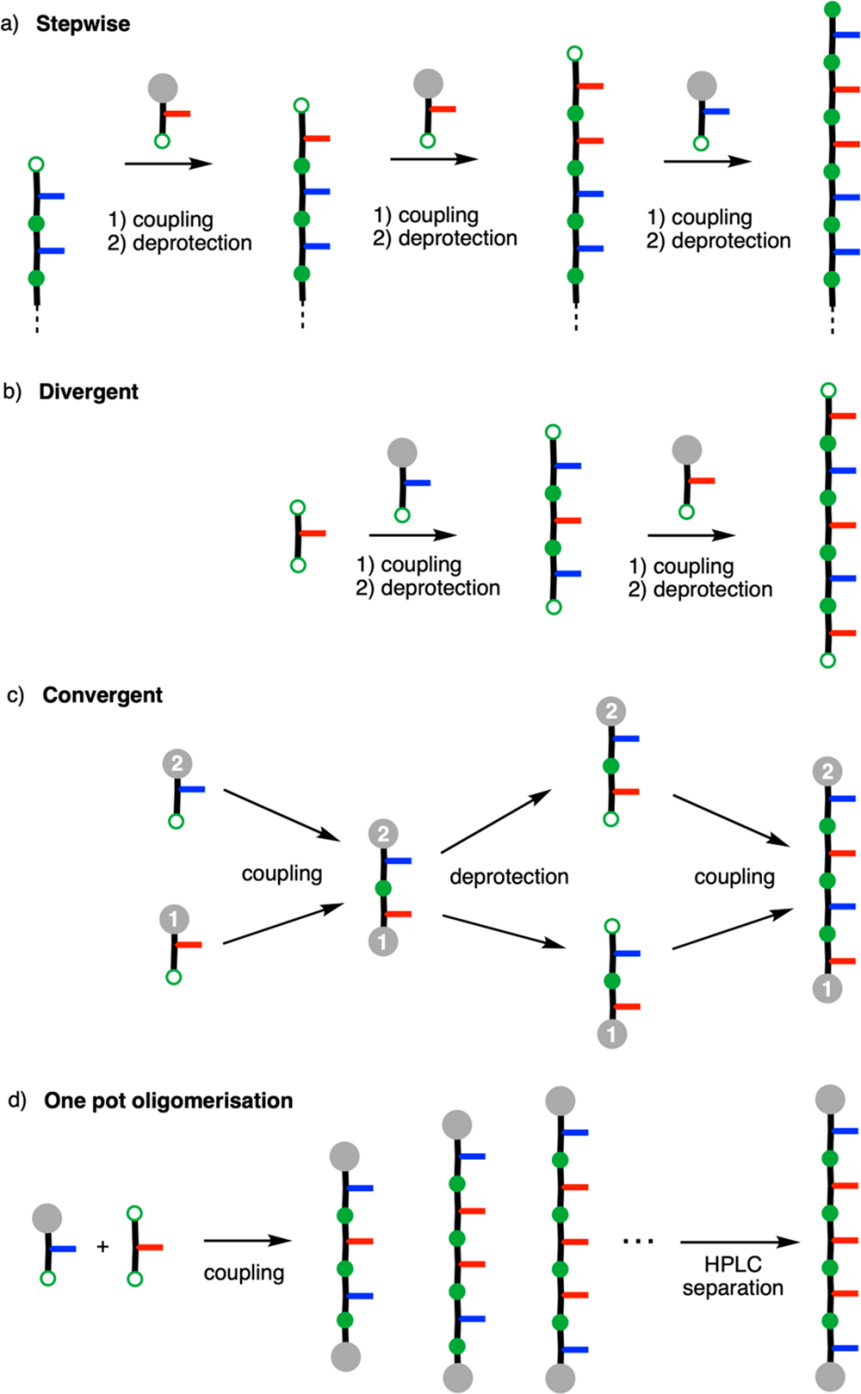
Synthetic strategies for oligomer preparation. (a) Stepwise. Protected monomers are added to one end of a growing oligomer and then deprotected. (b) Divergent. Protected monomers are added to both ends of a growing oligomer and then deprotected. (c) Convergent. Two orthogonally protected monomers are coupled, and the product is split in two fractions for removal of protecting group 1 or 2. (d) One pot oligomerization of monomers in the presence of a monofunctional chain stopper gives a mixture of oligomers that are separated by chromatography. Gray circles represent protecting groups, and open circles represent reactive sites for coupling reactions.

Four synthetic strategies have been used to obtain oligomers of different length and sequence (Figure 8). Figure 8a shows the classical stepwise approach: iterative coupling and deprotection reactions add monomers to a growing chain to afford an oligomer of any desired sequence. The divergent approach in Figure 8b is similar, except that the chain grows in two directions simultaneously, which gives products with 2-fold sequence symmetry. Figure 8c shows a convergent approach. Two orthogonally monoprotected monomers are coupled, and the product is split in half. Protecting group 1 is removed from one fraction, and protecting group 2 is removed from the other fraction. The cycle is repeated iteratively to double the length of the oligomer with each round of coupling. As with the divergent approach, only a subset of all possible sequences can be obtained with this method. Figure 8d shows how coupling of bifunctional monomers can be carried out to generate a mixture of oligomers in a single step. It is possible to control the average chain length by adding a monofunctional chain stopper, and the resulting mixture of oligomers was separated by HPLC.

## Duplex Formation by Complementary Homo-oligomers

**Figure 9:**
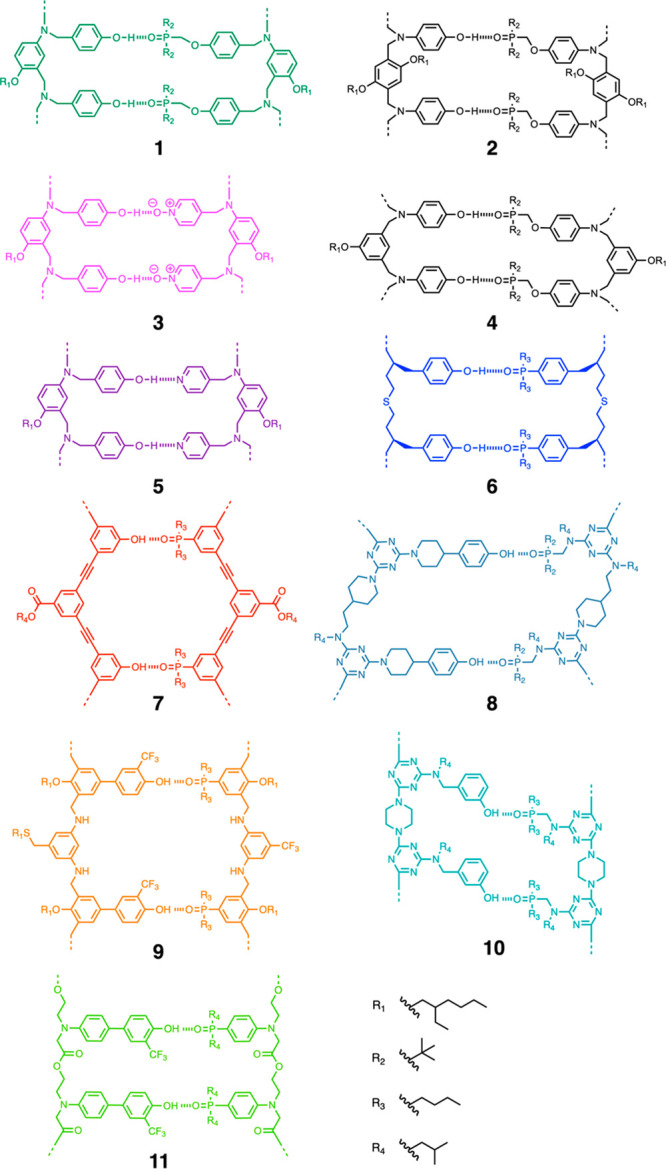
First two base-pairs of the duplex formed using oligomer architectures **1**–**11**.

**Figure 10:**
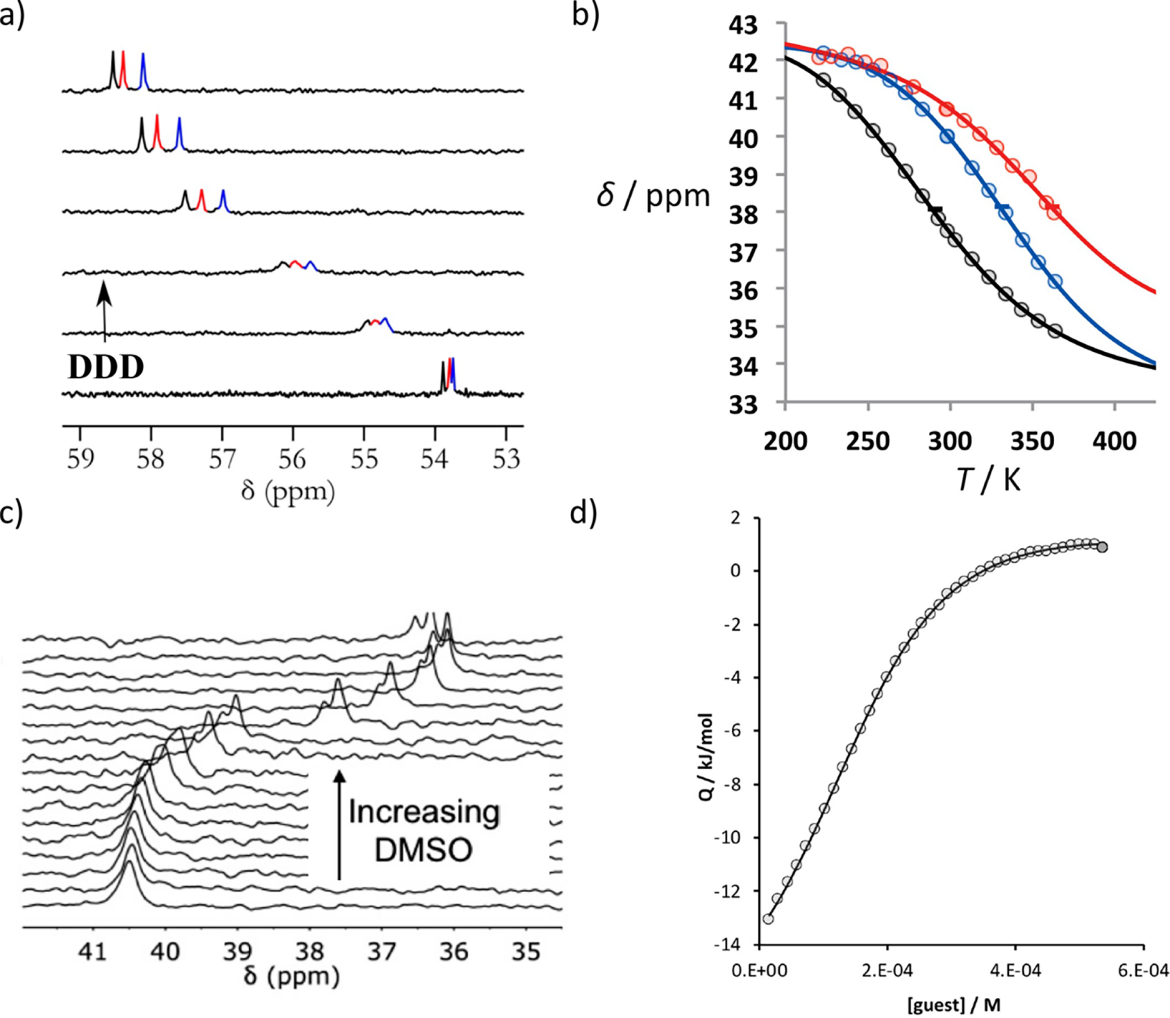
Different techniques used to measure association constants *K*_N_ between oligomers in toluene: (a) 162 MHz ^31^P NMR spectra for titration of **DDD** into **AAA** of oligomer **1** at 298 K in toluene-*d*_8_. (b) ^31^P NMR chemical shift of oligomer **6** plotted as a function of temperature for 1:1 mixtures of **A**·**D** (black), **AA**·**DD** (blue), and **AAA**·**DDD** (red) in toluene-*d*_8_. The horizontal bars show the transition melting temperatures, *T*_m,N_. (c) 202 MHz ^31^P NMR spectra for titration of DMSO-*d*_6_ into **DDD** and **AAA** of oligomer **7** at 298 K in toluene-*d*_8_. (d) ITC titration of **ADD** into **AAD** of oligomer **10** at 298 K in toluene.

Figure 9 shows the different oligomer architectures that we have investigated to date.^51,55−65^ In each case, homo-oligomers with up to four recognitions sites were synthesized, and the self-assembly properties were investigated using NMR titration, isothermal titration calorimetry (ITC), thermal denaturation, and denaturation using DMSO (Figure 10). ^1^H, ^31^P, and ^19^F NMR spectra provide direct information on the extent to which the recognition units are base-paired, because there are characteristic changes in chemical shift associated with H-bonding interactions. For example, Figure 10a shows data from a ^31^P NMR titration of the phenol 3-mer of oligomer **1** (**DDD**) into the corresponding phosphine oxide 3-mer (**AAA**) in toluene.^51^ All three of the signals due to the phosphine oxide groups have limiting complexation-induced changes in chemical shift of +5 ppm, which indicates that all of the phosphine oxide groups are engaged in H-bonding with a phenol in the complex. If all of the H-bond donor and acceptor recognition units in two complementary oligomers are fully H-bonded, then a fully base-paired duplex must have assembled.

**Figure 11:**
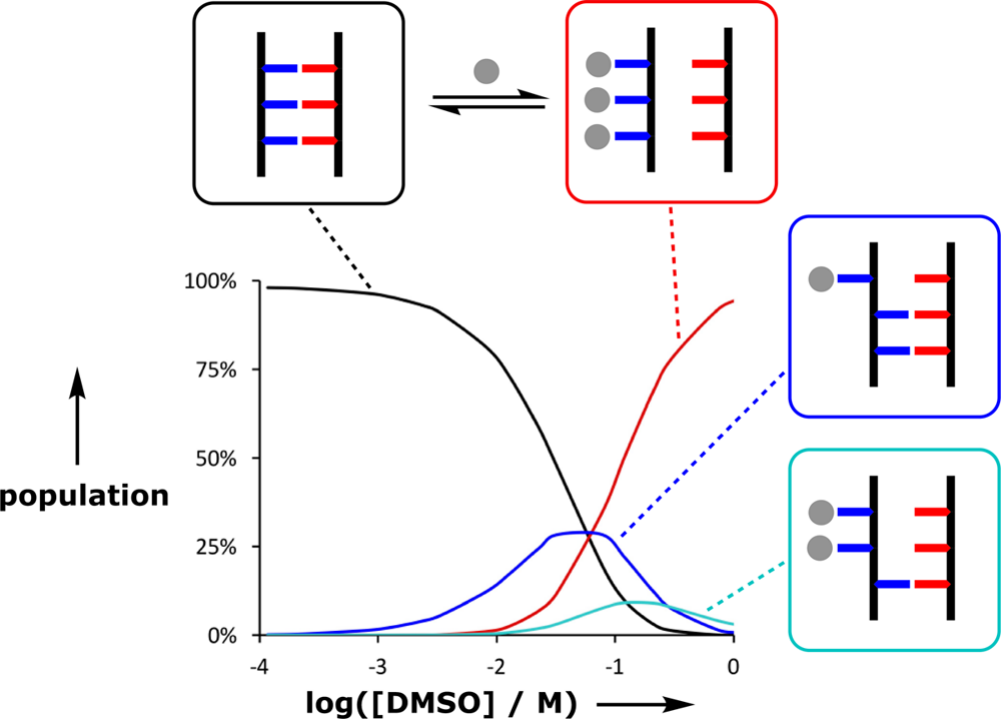
Population of intermediate states (blue boxes) in DMSO (gray balls) denaturation of the duplex formed between the phenol and phosphine oxide 3-mers of oligomer **7** in toluene.

NMR titrations were used to measure 1:1 association constants of up to 10^6^ M^–1^ for duplex formation between length complementary homo-oligomers in toluene. For more stable duplexes, different methods were required. It is possible to melt the duplexes using thermal denaturation (Figure 10b), but the temperature range required to observe the fully dissociated and fully assembled duplexes was too large for reliable use in the measurement of association constants. Denaturation with polar solvents proved more informative. When DMSO is titrated into a 1:1 mixture of two complementary oligomers, interactions between DMSO and the phenol recognition units compete with duplex formation. Figure 10c shows an example of a ^31^P NMR DMSO denaturation experiment.^61^ The decrease of 5 ppm in the chemical shifts of the ^31^P signals due to the phosphine oxide recognition groups is indicative of disruption of the phenol·phosphine oxide H-bonding interactions holding the duplex together at high DMSO concentrations. However, the denaturation data did not fit to a simple two-state, all-or-nothing isotherm, implicating partially denatured species. Figure 11 illustrates the speciation of intermediates populated in a duplex denaturation experiment. The intermediate in which only one of the base-pairs is broken (blue box) reaches a population of 25% before the fully denatured state (red box) starts to dominate.

The NMR spectra of the melamine oligomers **8** and **10** were complicated by slow rotation around the exocyclic nitrogen–triazine bonds. However, duplex formation in toluene is highly exothermic (−20 kJ mol^–1^ per phenol·phosphine oxide H-bond), so ITC proved more useful for determining association constants for these systems (Figure 10d).^1^

**Figure 12:**
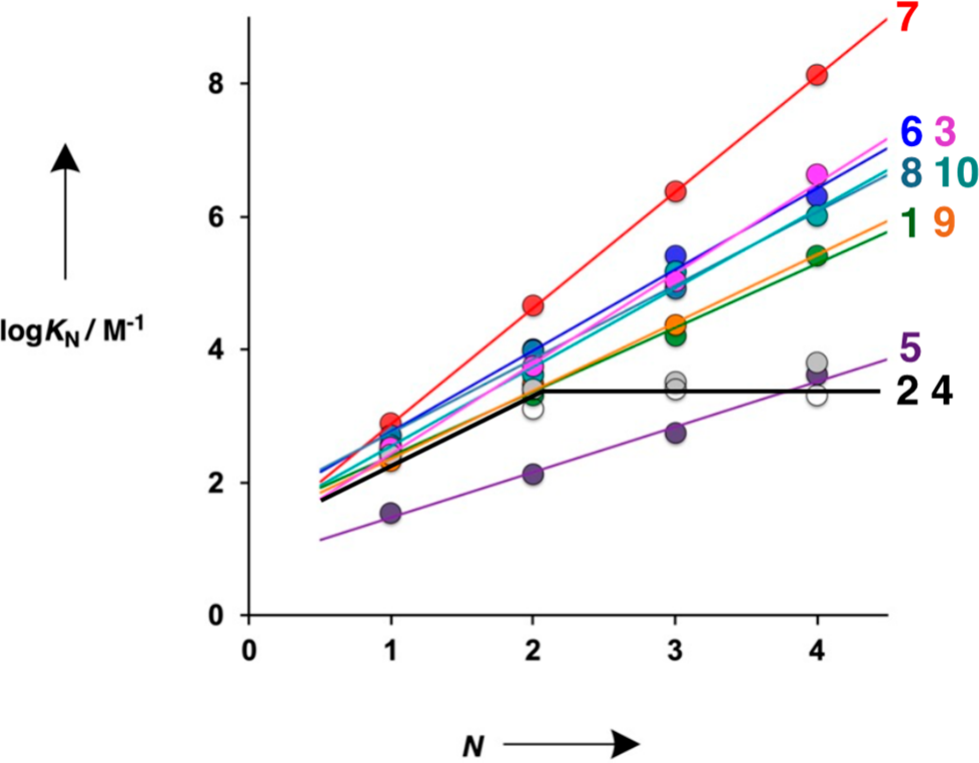
Association constants for duplex formation between length-complementary homo-oligomers in toluene (*K*_N_) plotted as a function of the number of recognition units N. The colors correspond to the oligomer numbers indicated.

The association constants for duplex formation between length complementary homo-oligomers (*K*_N_) are plotted in Figure 12 as a function of the number of base pairs (*N*) for each of the 11 architectures shown in Figure 9. The behavior of these systems is remarkably consistent. In most cases, there is an increase of an order of magnitude in the association constant for every base-pair added to the duplex. Oligomer **5** (purple) gives duplexes that are less stable than the other systems, because the pyridine recognition units are relatively weak H-bond acceptors (see Table 1). Oligomer **7** (red) gives duplexes that are somewhat more stable than the other architectures for reasons that will be discussed below. There are two oligomer architectures (**2** and **4**) that show quite different behavior: the 2-mers form duplexes, but no increase in stability is observed for 3-mers or 4-mers (black line), because the geometry of the backbone is not compatible with the propagation of longer duplexes.^58^

**Figure 13:**
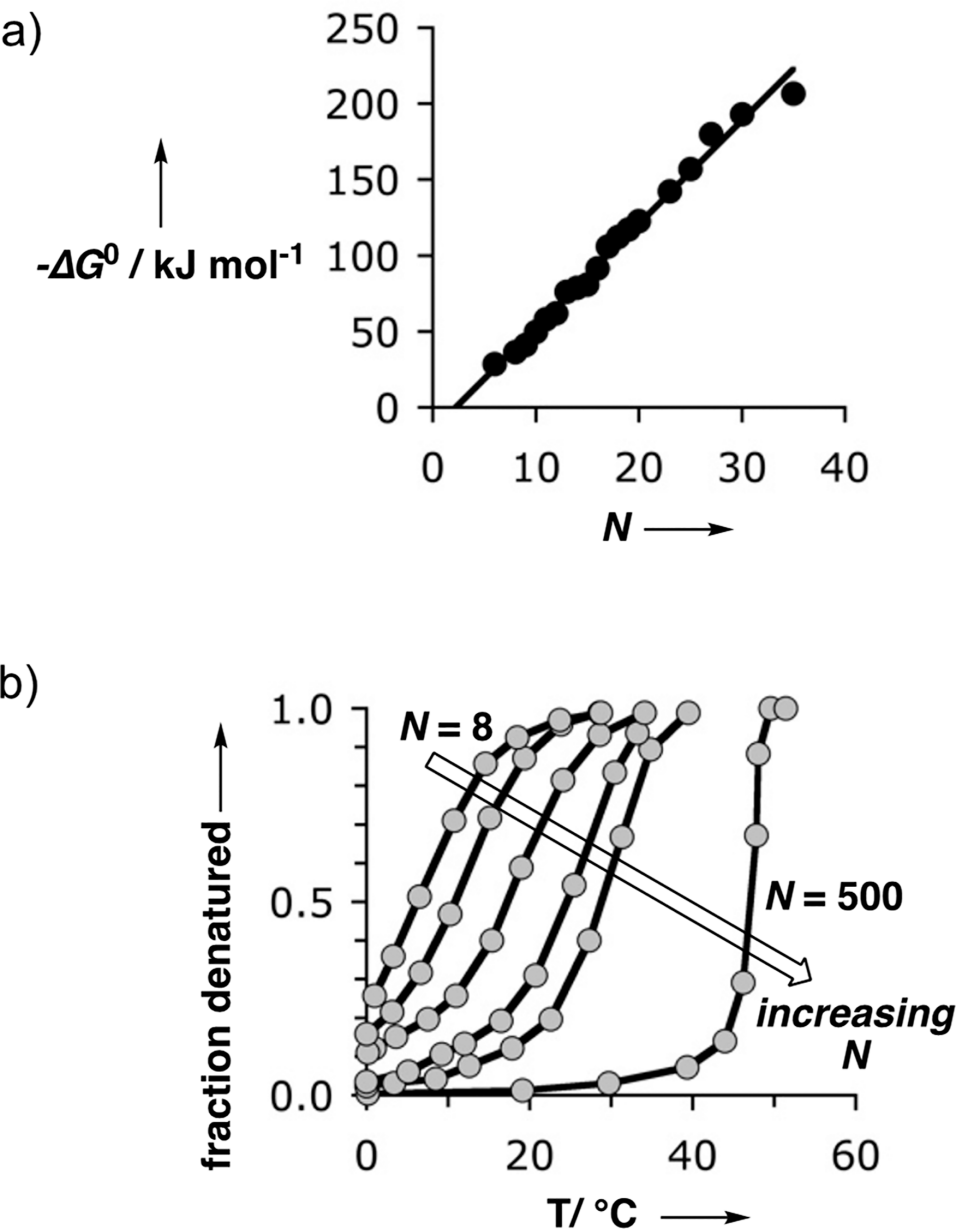
(a) Free energy of duplex formation plotted against the number of base pairs (*N*) for a range of DNA duplexes.^66^ (b) Thermal denaturation of oligo(rA)·oligo(rU) duplexes with a number of base pairs *N* = 8, 9, 11, 14, 18, and ca. 500.^67^

The results in Figures 11 and 12 have interesting implications for understanding the nature of the cooperativity associated with duplex formation. The H-bonding interactions between two oligomers within an assembled duplex operate cooperatively, so that all of the base-pairs are fully bound, and the stability of the duplex increases uniformly with the number of interactions. This behavior is precisely the same as that observed for nucleic acid duplexes, where stability increases by an order of magnitude for every base-pair added (Figure 13a). However, the denaturation experiments show that the transition from single-stranded oligomers to duplex is not an all-or-nothing process (Figure 11), and melting of the synthetic duplexes takes place over a relatively wide temperature window (Figure 10c) compared with nucleic acid duplexes in Figure 13b.

The linear increase in duplex stability with chain length shown in Figure 12 indicates that the value of EM_d_ is constant and does not change with the number of base-pairs. The values of EM_d_ can therefore be determined from the slopes of the lines in Figure 12 using eq 2.
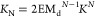
2where the factor of 2 accounts for the degeneracy of the duplex formed by two homo-oligomers.

Table 2 lists the reference association constants for the intermolecular base-pairing interaction *K*, the values of EM_d_, and the product *K* EM_d_, which quantifies the magnitude of the chelate cooperativity associated with duplex formation. The values of EM_d_ all fall in the range 10–100 mM, and there is limited variation with the structure of the backbone. For example, the values of EM_d_ for the most flexible backbone **6** and the most rigid backbone **7** are very similar (25 and 49 mM). The variation in chelate cooperativity (*K* EM_d_) is larger. The highest values are found for oligomers **9** and **11**, which use the strongest H-bond donor, 2-(trifluoromethyl)phenol, and the lowest value is observed for oligomer **5**, which uses the weakest H-bond acceptor, pyridine. This result demonstrates that a straightforward strategy for ensuring efficient duplex formation is to choose a base-pair with a very strong H-bond.

**Table 2:** Base-Pair Association Constants (*K*), Effective Molarities for Intramolecular Interactions in Duplex Assembly (EM_d_) and Chelate Cooperativity Parameters (*K* EM_d_) Measured in Toluene at 298 K for the Oligomers Shown in Figure 9^a^

oligomer	donor	acceptor	*K*, M^–1^	EM_d_, mM	*K* EM_d_
**1**	phenol	phosphine oxide	350	16	5
**3**	phenol	pyridine *N*-oxide	330	39	13
**5**	phenol	pyridine	34	84	3
**6**	phenol	phosphine oxide	500	25	13
**7**	phenol	phosphine oxide	760	49	37
**8**	phenol	phosphine oxide	630	33	12
**9**	2-(trifluoromethyl)phenol	phosphine oxide	3160	34	110
**10**	phenol	phosphine oxide	360	29	10
**11**	2-(trifluoromethyl)phenol	phosphine oxide	3900	19	75

a Oligomers **2** and **4** do not form duplexes with more than two base-pairs.

**Figure 14:**
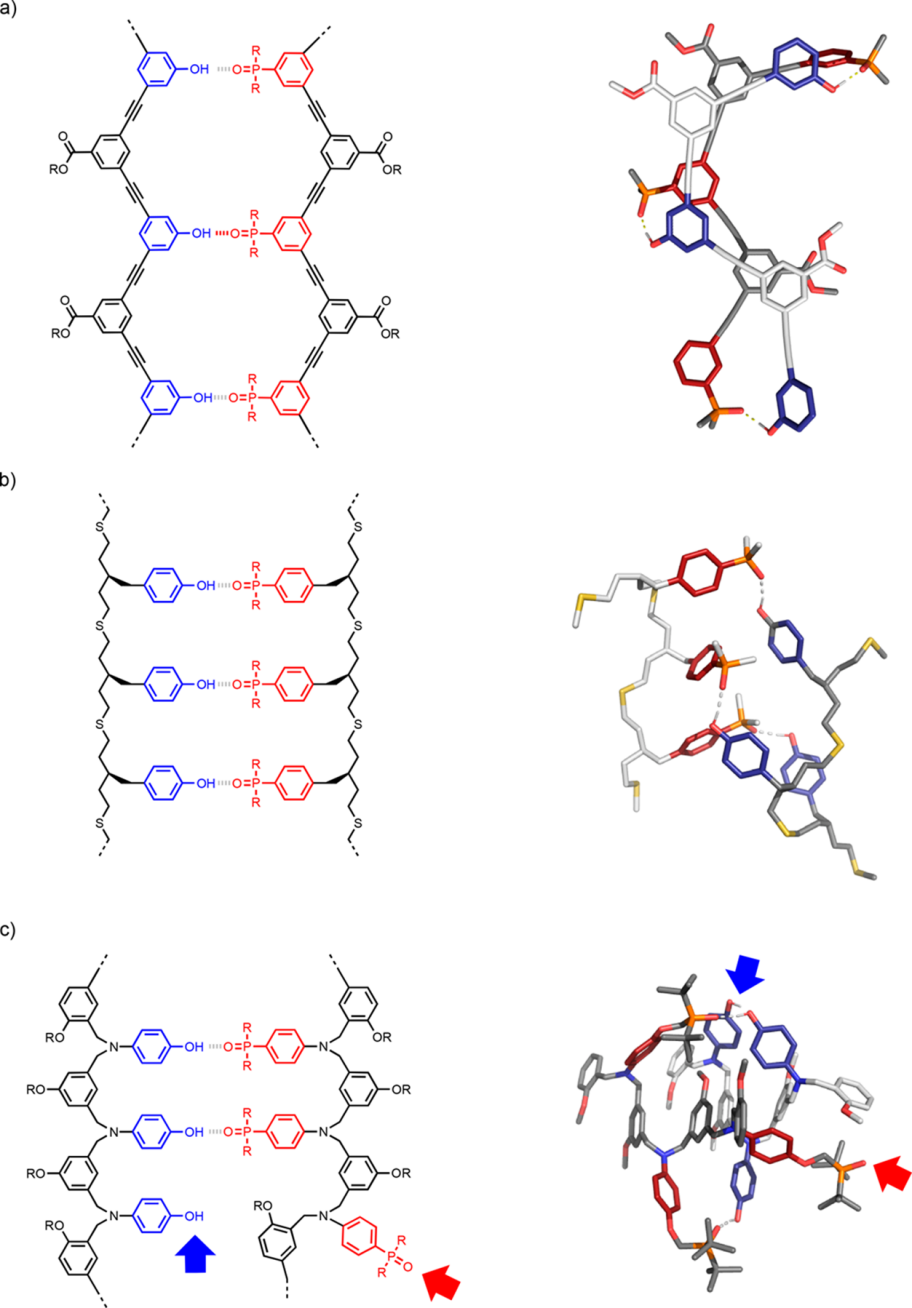
Lowest energy molecular mechanics structures of 3-mer duplexes formed by homo-oligomers with different backbones: (a) **7**, (b) **6**, (c) **4**. Arrows in panel c highlight the unpaired phenol (blue) and the unpaired phosphine oxide (red). R represents solubilizing groups, which were replaced by methyl groups in the calculations.

The two oligomers missing from Table 2 are **2** and **4**, which do not form extended duplexes with more than two base-pairs. Molecular mechanics calculations provide some insight into the conformation properties of different backbones. Figure 14 compares the lowest energy structures of the 3-mer duplexes formed by **4**, **6**, and **7**. For **6** and **7**, the most stable conformations are the fully H-bonded duplexes, in agreement with experiment.^61,63^ In contrast, the lowest energy conformation illustrated for oligomer **4** is the partially bound duplex where only two H-bonds are formed.^58^ Similar results were obtained for oligomer **2**, which suggests that molecular mechanics may provide a useful tool for filtering out backbones that are not compatible with duplex assembly. For a very flexible backbone such as **6**, it will always be possible to find a conformation in which all of the base-pairs can be formed. For more rigid backbones, the conformational properties of the backbone play a critical role. Duplex assembly will occur for rigid backbones where there is precise geometric complementarity, such as **7**.

## Folding in Mixed Sequence Oligomers

**Figure 15:**
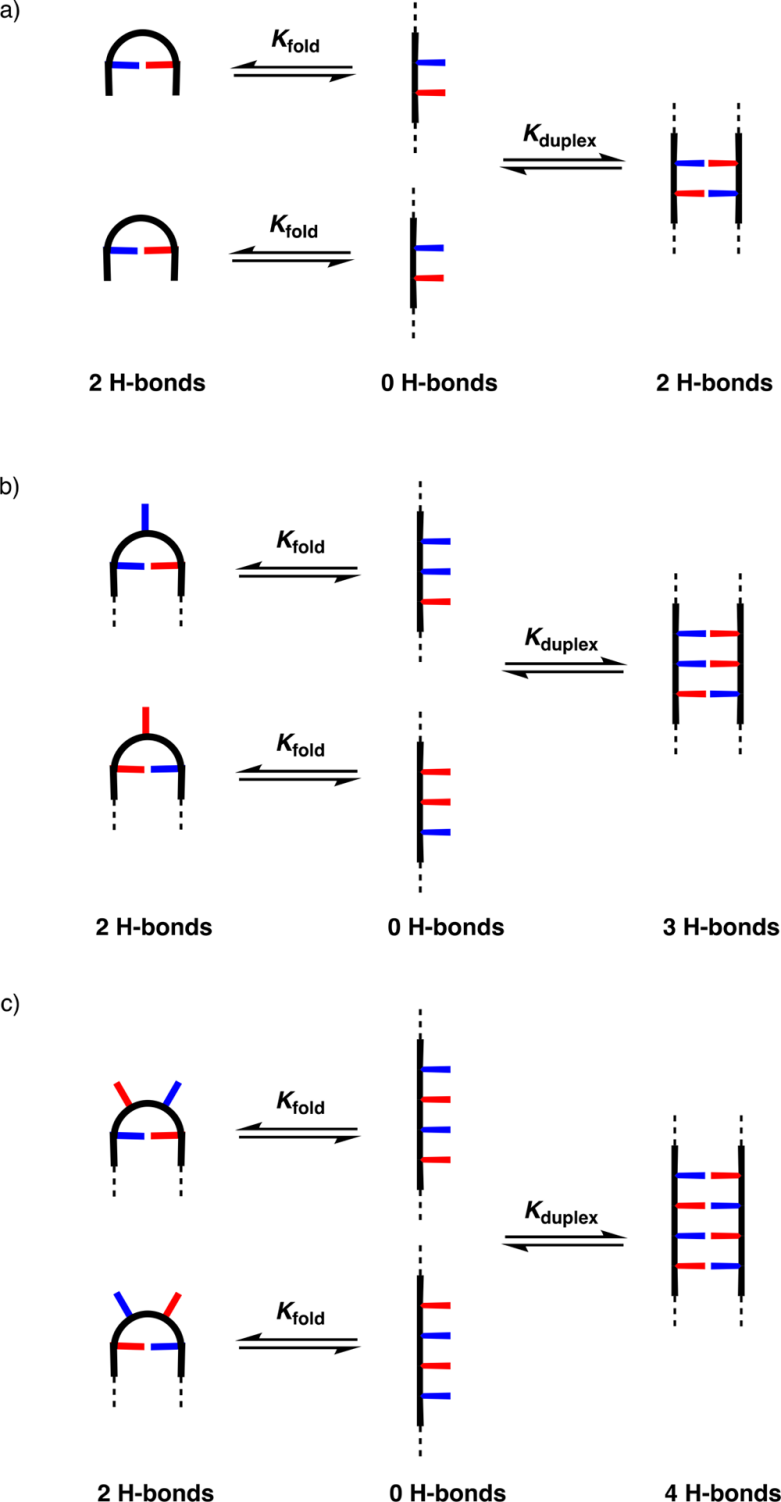
Folding equilibria for mixed sequence oligomers: (a) 1,2-folding, (b) 1,3-folding, (c) 1,4-folding.

When two different recognition modules are present in the same oligomer, intramolecular base-pairing becomes possible. These folding equilibria compete with the intermolecular interactions that lead to duplex formation. There are different ways in which an oligomer could fold on itself, depending on sequence. We have investigated the simplest folding patterns, which are schematically represented in Figure 15. Once a mixed sequence oligomer is long enough, it will always be capable of folding, indeed sequence-programmed folding is an important property of biopolymers. However, if short oligomers form very stable folds, duplex formation becomes highly unlikely for most sequences. For example, Figure 15a shows that duplex formation results in no net gain in the number of base-pairing interactions, if two neighboring recognition modules in a sequence can interact to make a 1,2-fold.

Figure 15 shows that as the size of the loop formed in the folding process becomes longer there is an increase in the number of base-pairing interactions that favor the duplex over folded structures. For Watson–Crick base-pairing in nucleic acids, none of the three folding processes shown in Figure 15 is observed, but 1,5-folding leads to the characteristic motif known as a stem-loop.^68^ There is a mismatch between the cross-section of the Watson–Crick base-pair (10 Å from one backbone to the other) and the length of the backbone (5 Å between bases), which means that a minimum of three bases must be looped out for the backbone to fold. This kind of geometric constraint is not imposed by single H-bond base-pairs, so folding is more prevalent in the synthetic oligomers.

The self-assembly properties of self-complementary 2-mers with one H-bond donor (**D**) and acceptor (**A**) can be used to assess the 1,2-folding propensity of an oligomer.^2^Equation 3 shows the relationship between the self-association constant measured for an **AD** 2-mer and the equilibria shown in Figure 15a.
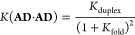
3where the value of *K*_duplex_ can be estimated from the association constant measured for the corresponding **AA**·**DD** duplex.

**AD** 2-mers of each of the architectures shown in Figure 9 were studied using NMR dilution experiments. For oligomers **3**, **5**, **7**, **10**, and **11**, no evidence of 1,2-folding was detected, and the association constant measured for **AD·AD** was comparable to the **AA**·**DD** duplex. The other oligomers populate the 1,2-folded state to a significant extent, reducing the self-association constant measured for **AD** by orders of magnitude compared with the corresponding **AA**·**DD** duplex. Values of *K*_fold_ for these oligomers and the effective molarities EM_f_ for the intramolecular interaction are summarized in Table 3. The NMR chemical shifts observed for single-stranded species in dilute solutions support the conclusions based on measurement of association constants. For example, the phenol OH signal in the ^1^H NMR spectrum of a dilute solution of the **AD** 2-mer of oligomer **9** appears at 11.2 ppm, which represents a downfield shift of +6 ppm compared with the corresponding phenol monomer and indicates the presence of an intramolecular H-bond.^59^ The value of *K*_fold_ for this oligomer in Table 3 indicates that the 1,2-folded state is 95% populated in the single-stranded species.

**Table 3:** Equilibrium Constants for 1,2-Folding of **AD** 2-mers (*K*_fold_) and Effective Molarities for the Intramolecular Interaction (EM_f_)

oligomer	*K*_fold_	EM_f_, mM
**1**	2.2	7
**2**	0.7	3
**4**	3.6	15
**6**	4.1	7
**8**	11	18
**9**	19	7

**Figure 16:**
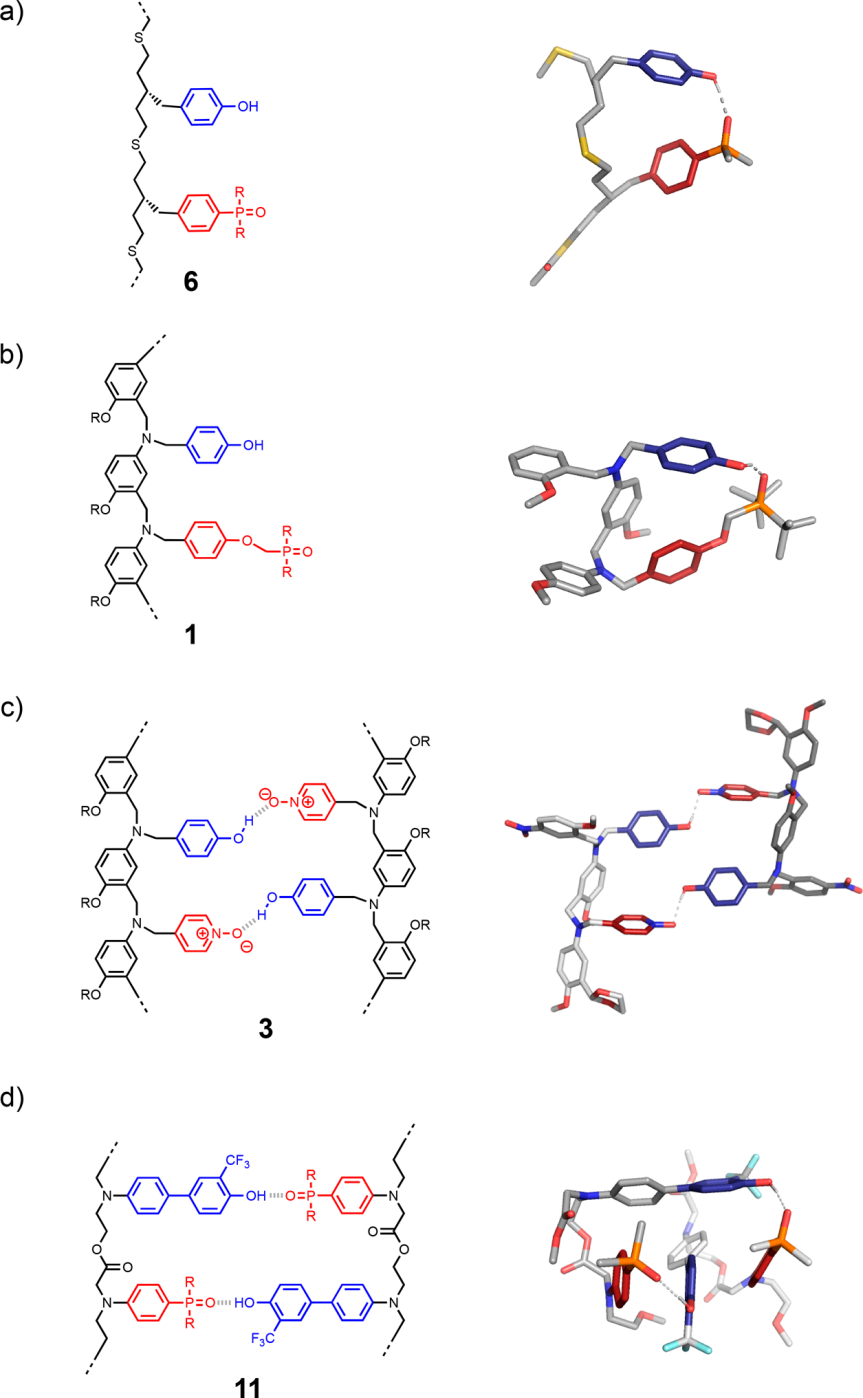
Influence of oligomer architecture on 1,2-folding. (a) A flexible backbone leads to folding. Molecular mechanics structure of the lowest energy conformation of the **AD** 2-mer of **6**. (b) A flexible base-pair leads to folding. Molecular mechanics structure of the lowest energy conformation of the **AD** 2-mer of **1**. (c) A more rigid architecture favors duplex formation. X-ray crystal structure of the **AD**·**AD** duplex of **3**. (d) A geometric mismatch between base-pair cross-section and backbone length prevents 1,2-folding. Molecular mechanics structure of the lowest energy conformation of the **AD**·**AD** duplex of **11**. R represents solubilizing groups, which were replaced by methyl groups in the calculations.

Conformational flexibility is a key determinant of folding propensity. Oligomers with flexible backbones, such as **6**, tend to fold (Figure 16a), whereas rigid backbones like **7** do not. Similarly, attachment of the recognition units to the backbone via flexible linkers, as in **1**, increases folding propensity (Figure 16b). Oligomer **3** shares the same backbone as **1**, but the base-pairing system is more rigid, and folding is abolished. Figure 16c shows the X-ray crystal structure of the **AD**·**AD** duplex of **3**. Another strategy for avoiding 1,2-folding is to make the base-pair longer than the backbone that connects two bases. Figure 16d shows the **AD**·**AD** duplex of **11**, where the extended base-pairing system allows a more flexible backbone to be employed without any 1,2-folding.

**Figure 17:**
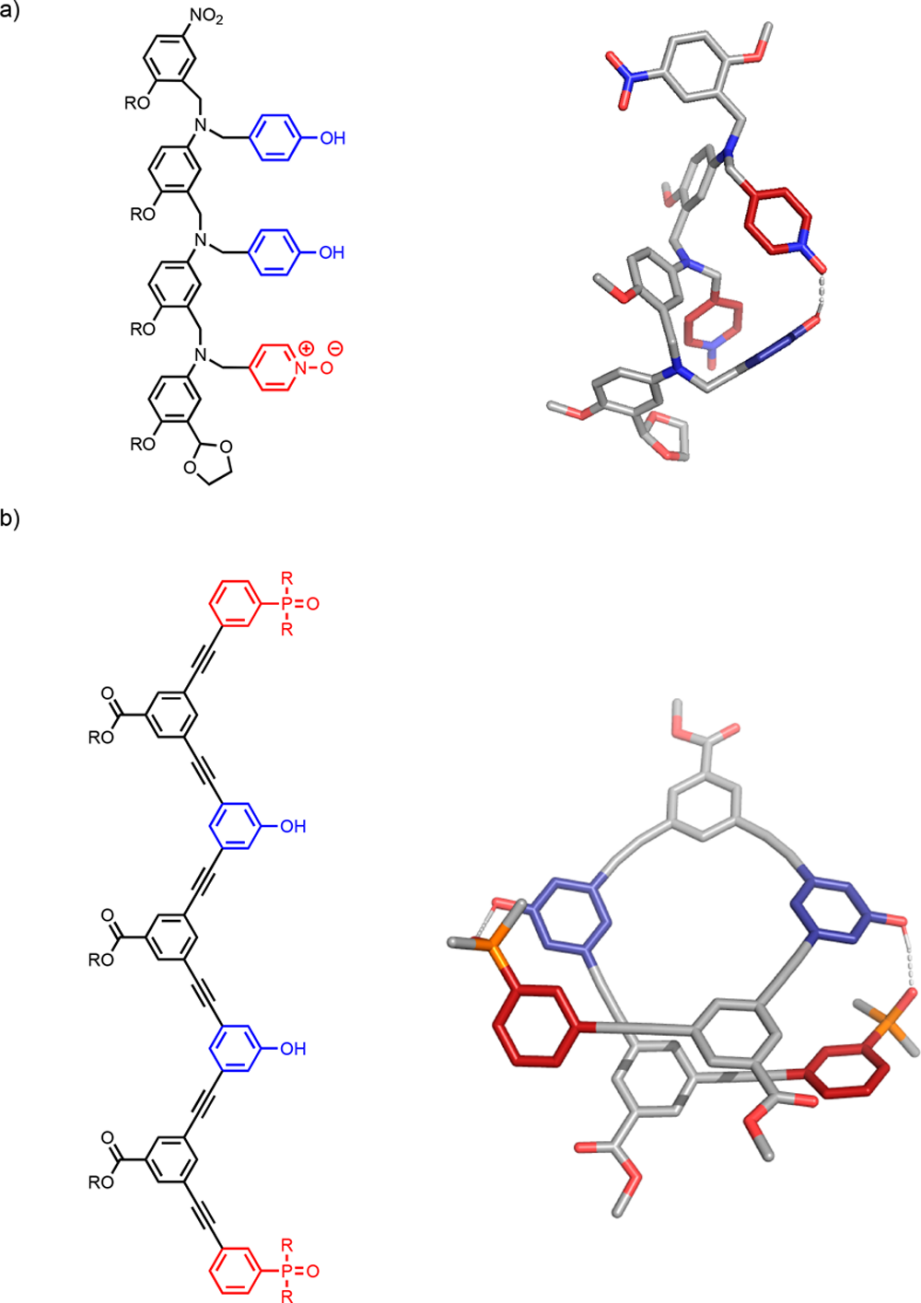
Oligomer architectures where 1,3-folding occurs. (a) The **DDA** 3-mer of **3**, and the molecular mechanics structure of the lowest energy conformation. (b) The **ADDA** 4-mer of **7**, and the molecular mechanics structure of the lowest energy conformation. R represents solubilizing groups, which were replaced by methyl groups in the calculations.

A similar approach can be used to investigate the 1,3-folding properties of mixed sequence oligomers. There are two 3-mer sequences with complementary chain ends that have the potential for 1,3-folding (**AAD** and **ADD**). Comparison of the association constant for formation of the **AAD**·**ADD** duplex with the corresponding **AAA**·**DDD** duplex quantifies the 1,3-folding propensity. The stabilities of the duplexes formed by all sequence-complementary 3-mers were measured for oligomer architectures **3** and **10**.^1,57^ No evidence of intramolecular folding was detected for any of the 3-mer sequences of oligomer **10**, but 1,3-folding was found to compete with duplex formation for oligomer **3**. Molecular mechanics confirms that the lowest energy conformation for a single strand of the **DDA** 3-mer of oligomer **3** has an intramolecular H-bond between the terminal recognition modules (Figure 17a). Similar experiments show that 1,3-folding also occurs for oligomer **7** (Figure 17b shows two intramolecular H-bonds in doubly 1,3-folded **ADDA**).^62^

## Sequence-Selectivity of Duplex Formation

**Figure 18:**
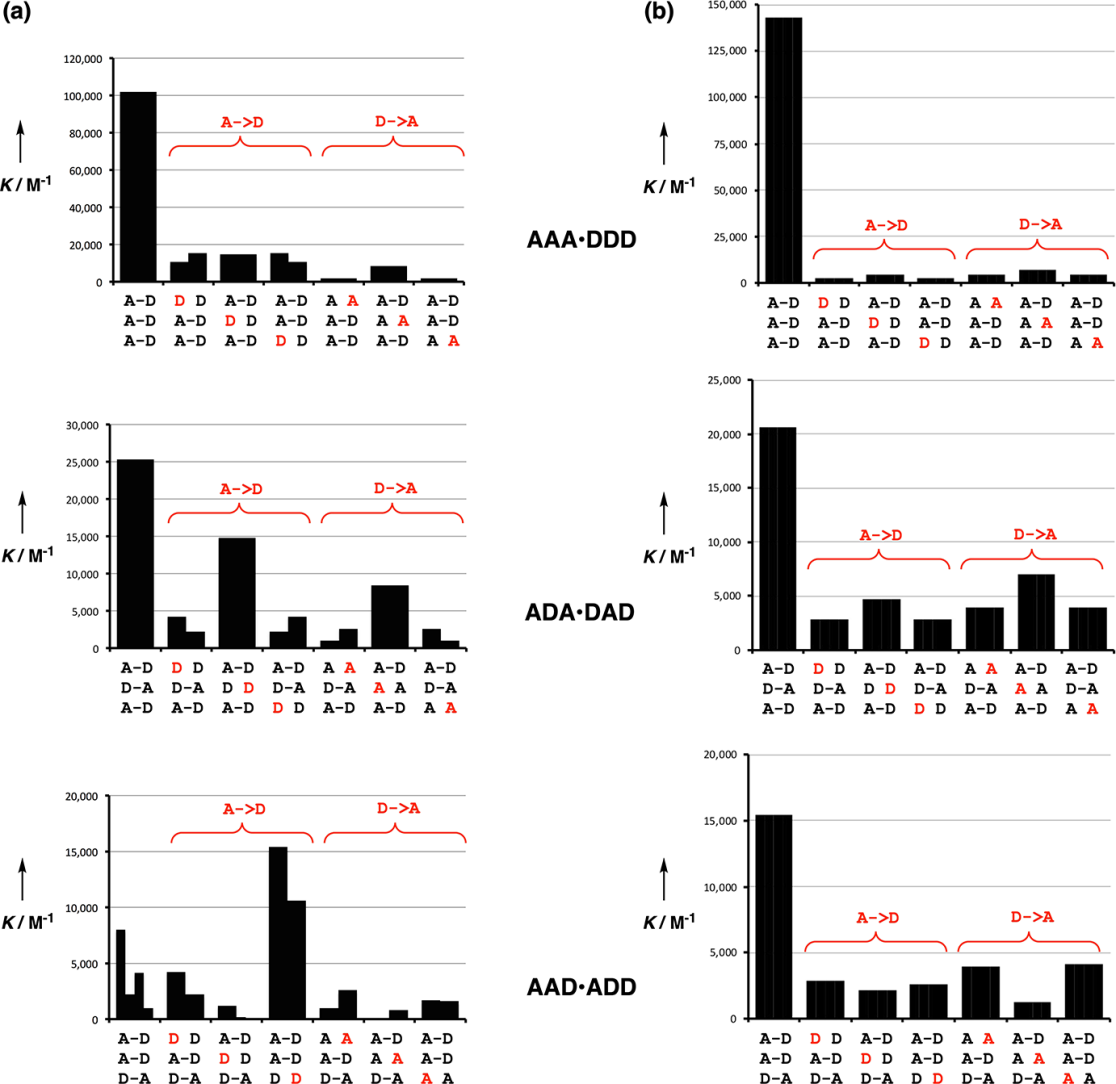
Single site mismatch analysis showing the effect of single **A** → **D** or **D** → **A** mutation (red) on the stabilities of sequence-complementary 3-mer duplexes for (a) oligomer **3** and (b) oligomer **10**. The first entry in each bar chart shows the association constant for formation of the sequence-complementary duplex.

Interactions between all pairwise combinations of 3-mer sequences have been measured for oligomers **3** and **10**.^1,57^ The results are illustrated using a single base mismatch analysis in Figure 18. There are three sequence-complementary 3-mer duplexes, **AAA**·**DDD**, **ADA**·**DAD**, and **AAD**·**ADD**, and the first entry in each bar chart in Figure 18 shows the association constant for formation of the duplex from the two complementary strands. The other entries show the effects of all possible single base mismatches, i.e., changing one **A** to a **D**, or one **D** to an **A**). For oligomer **3**, some of the mismatch duplexes are more stable than the sequence-complementary duplex (Figure 18a), but for oligomer **10**, high fidelity duplex formation is observed, and all of the mismatch duplexes are significantly less stable than the sequence-complementary duplexes (Figure 18b). The biggest difference is observed for the **AAD**·**ADD** duplexes, where 1,3-folding competes with duplex formation for both of the single-stranded oligomers in **3**. The lack of any competing folding equilibria in oligomer **10** leads to reliable sequence-selectivity for this architecture.

## Conclusion

The experiments described here allow us to draw some general conclusions about the design of sequence-selective duplex forming oligomers.

### Recognition Module

The effective molarity for duplex formation (EM_d_) is in the range 10–100 mM for almost all of the systems prepared to date, regardless of the properties of the backbone. As a consequence, the association constant for intermolecular base-pair formation between the recognition modules should be at least 100 M^–1^ in order to ensure duplex assembly. Systems which use recognition units or solvents that lead to lower association constants are unlikely to form duplexes, no matter how carefully the backbone is designed.

### Backbone Module

The choice of backbone has a minimal impact on the value of EM_d_, so pretty much any pair of homo-oligomers equipped with complementary recognition units will form a duplex. However, the conformational properties of the backbone have a significant impact on the folding propensity of mixed sequence recognition-encoded oligomers, and intramolecular folding of single-stranded oligomers has a direct impact on the sequence-selectivity of duplex formation. High fidelity duplex formation was only observed for a backbone that was sufficiently rigid to prevent both 1,2-folding between adjacent bases in the sequence and 1,3-folding.

### Synthesis Module

The preparation of polymeric sequences requires efficient coupling chemistry that does not compete with the noncovalent interactions used in the recognition modules. However, one of the practical challenges that has emerged is the development of robust synthetic routes for the preparation of complementary trifunctional monomer building blocks on a sufficiently large scale for use in oligomer synthesis.

## Outlook

**Figure 19:**
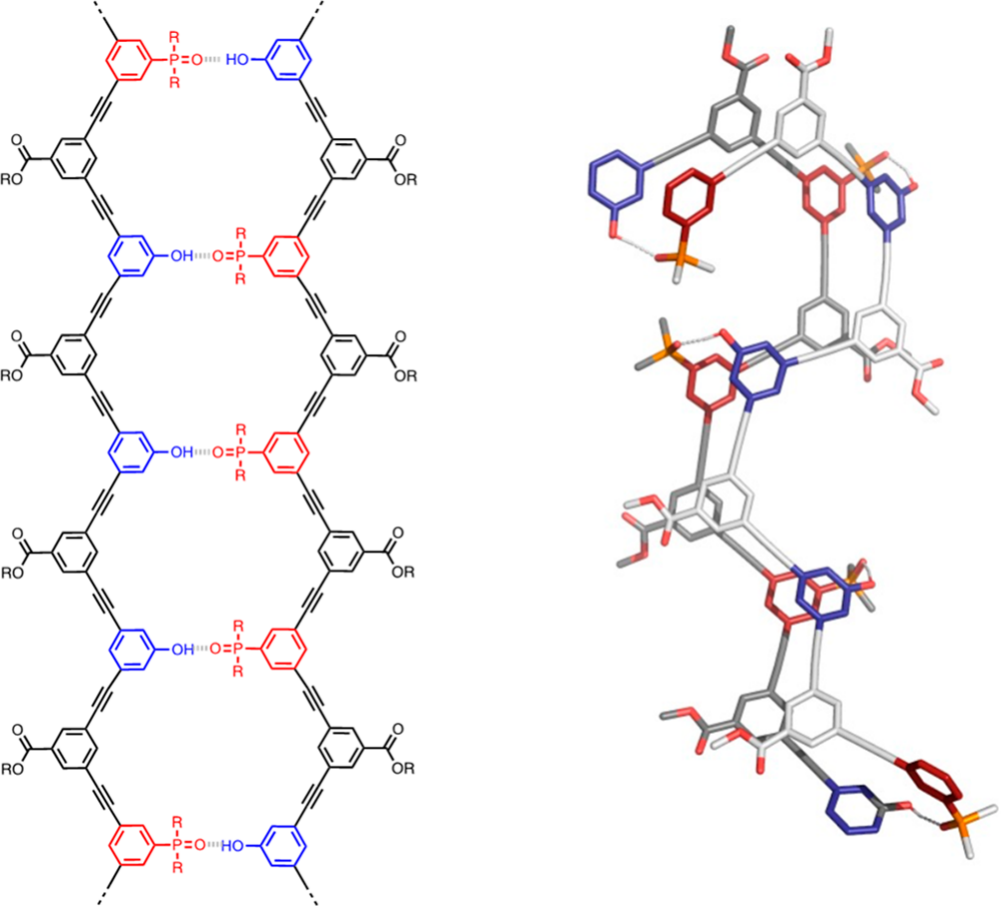
Molecular mechanics structure of the lowest energy conformation of the **ADDDA**·**DAAAD** duplex of oligomer **7**. R represents solubilizing groups, which were replaced by methyl groups in the calculations.

Most of the examples discussed in this review describe relatively short sequences that were used to establish the basic principles of synthetic duplex assembly. The next step is to investigate how well this behavior translates to longer oligomers. The synthetic strategy illustrated in Figure 8d has been used to prepare mixed sequences of oligomer **7** up to seven monomer units long.^62^ Sequence-complementary oligomers formed duplexes with significantly enhanced stability compared with the shorter oligomers. Figure 19 shows the structure of the duplex formed by two 5-mers, **ADDDA**·**DAAAD**, which suggests that observations made on short oligomers should translate well to longer sequences.

**Figure 20:**
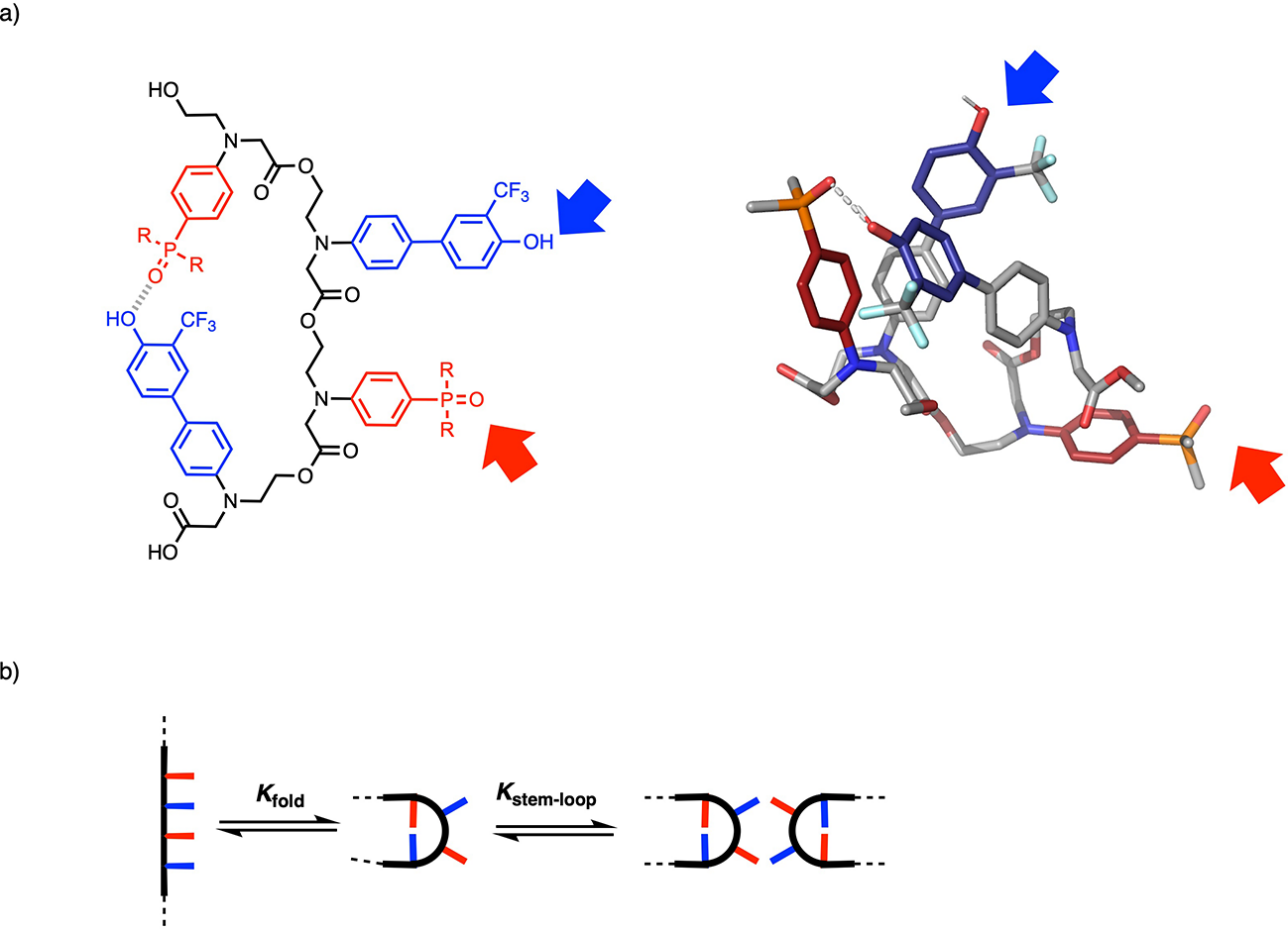
Self-assembly of a kissing stem-loop. (a) The **ADAD** 4-mer of **11**, and the molecular mechanics structure of the lowest energy conformation of the single-strand. Arrows indicate unpaired recognition sites. (b)1,4-Folding and stem-loop equilibria.

The parallels between the properties of these synthetic oligomers and biopolymers are not limited to duplex formation, and synthetic recognition-encoded oligomers have the potential to recapitulate many of the functions found in biological systems: self-assembly, substrate recognition, catalysis, and replication. For example, the **ADAD** 4-mer of oligomer **11** self-assembles into the kissing stem-loop structure shown in Figure 20.^65^ There is an intramolecular 1,4-folding interaction between the two terminal bases, and the two inner bases in the sequence dimerize this loop structure via intermolecular H-bonds. This arrangement is one of the key structural elements of folded RNA and suggests that these synthetic oligomers are likely to show sequence-dependent self-assembly properties that are similar to the biopolymer.

**Figure 21:**
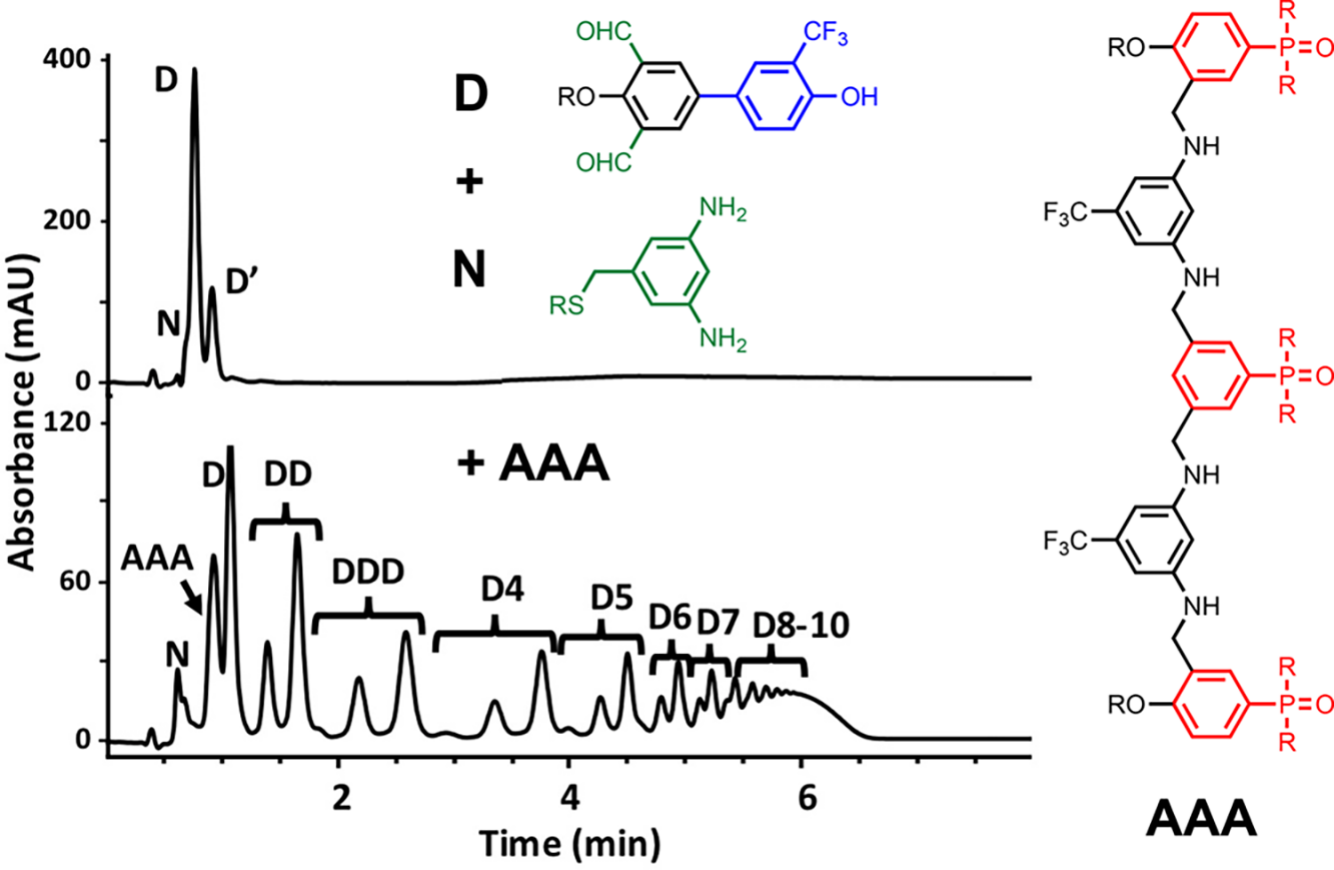
Catalysis of the imine condensation between **D** and N by the **AAA** 3-mer of oligomer **9**. HPLC traces of the crude reaction mixtures show that in the absence of **AAA** (top), little reaction takes place, but in the presence of **AAA** (bottom), rapid polymerization takes place to give a mixture of **D** oligomers.

The **AAA** 3-mer of oligomer **9** was found to have catalytic properties that resemble enzyme catalysis.^60^Figure 21 illustrates the imine polymerase activity found when **AAA** was added to a mixture of a diamine (**N**) and a dialdehyde equipped with a trifluoromethylphenol recognition unit (**D**). The reaction between **N** and **D** is slow, but rapid polymerization takes place in the presence of **AAA**. The presence of the phenol recognition unit on the monomer is essential for catalysis, which suggests that H-bonding interactions with **AAA** are important. Although the precise mechanism is not known, neither **A** or **AA** were active, and 2-trifluoromethylphenol was found to be a competitive inhibitor of **AAA**, consistent with enzyme-like catalytic activity.

**Figure 22:**
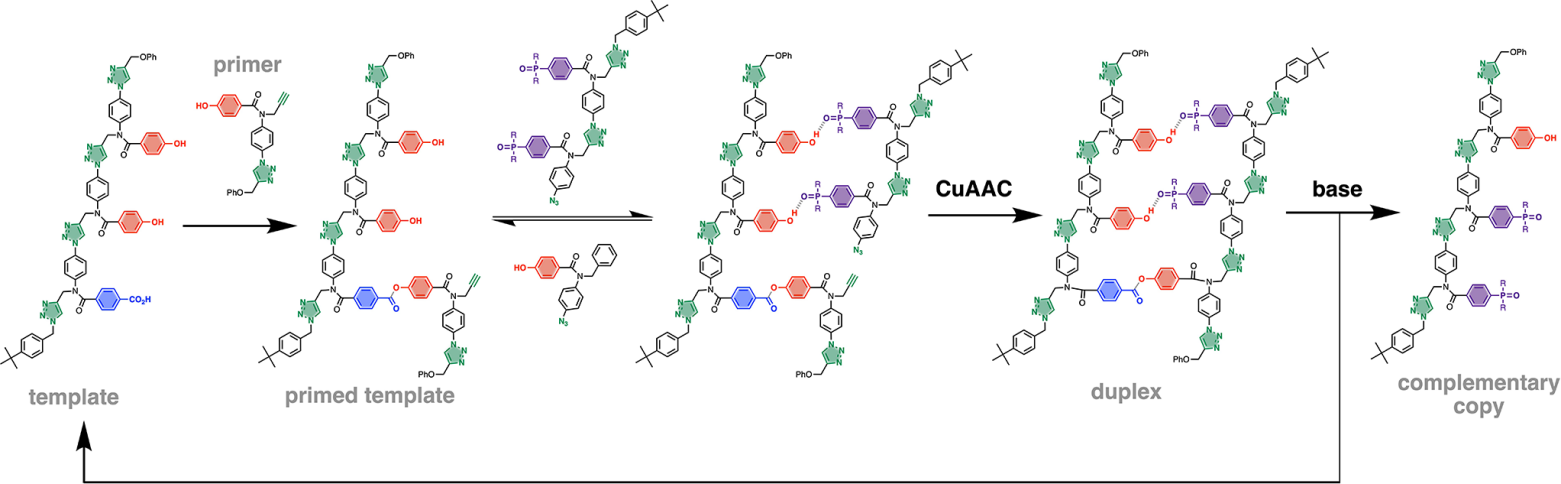
Template-directed replication of a mixed sequence oligomer. The primer was first loaded onto the template via covalent base-pairing using ester chemistry. H-bonding interactions with the phosphine oxide 2-mer led to selective incorporation in a CuAAC reaction. Hydrolysis of the ester base-pair in the resulting duplex gave the complementary copy strand and regenerated template.^69^

We have also begun preliminary studies into replication of the sequence information encoded in synthetic oligomers. The template shown in Figure 22 is a mixed sequence oligomer with an oligotriazole backbone, which was synthesized using copper catalyzed alkyne azide cycloaddition chemistry (CuAAC).^69^ This system was used in template-directed synthesis to transfer the sequence information to a daughter copy strand. First, a covalent primer equipped with an alkyne was attached to the template by esterification, i.e., a covalent base-pair between phenol and benzoic acid recognition units. When this primed template was exposed to two different azides under CuAAC conditions, the phosphine oxide 2-mer was selectively coupled with the primer, due to H-bonding interactions with the phenol recognition units on the template, to give the mixed covalent/noncovalent duplex shown in Figure 22. Finally, hydrolysis of the ester base-pair regenerated the template and released the copy strand, the sequence complement of the original template. Such replication processes could ultimately form the basis for molecular evolution of synthetic polymers.
